# Vertical-type two-dimensional hole gas diamond metal oxide semiconductor field-effect transistors

**DOI:** 10.1038/s41598-018-28837-5

**Published:** 2018-07-13

**Authors:** Nobutaka Oi, Masafumi Inaba, Satoshi Okubo, Ikuto Tsuyuzaki, Taisuke Kageura, Shinobu Onoda, Atsushi Hiraiwa, Hiroshi Kawarada

**Affiliations:** 1 Faculty of Science and Engineering, Waseda University, 3-4-1, Ohkubo, Shinjuku-ku, Tokyo 169-8555 Japan; 2 Research Organization for Nano & Life Innovation, Waseda University, 513 Waseda-tsurumaki, Shinjuku-ku, Tokyo 162-0041 Japan; 3 Institute of Materials and Systems for Sustainability, Nagoya University, Furo-cho, Chikusa-ku, Nagoya 464-8603 Japan; 4 National Institutes for Quantum and Radiological Science and Technology, 1233 Watanuki-cho, Takasaki-shi, Gunma 370-1292 Japan; 5 Institute of Materials and Systems for Sustainability (Tokyo Branch), Nagoya University, Bldg. 120-5 (Waseda University), 513 Waseda-tsurumaki, Shinjuku-ku, Tokyo 162-0041 Japan; 6 The Kagami Memorial Laboratory for Materials Science and Technology, Waseda University, 2-8-26 Nishiwaseda, Shinjuku-ku, Tokyo 169-0051 Japan

## Abstract

Power semiconductor devices require low on-resistivity and high breakdown voltages simultaneously. Vertical-type metal-oxide-semiconductor field-effect transistors (MOSFETs) meet these requirements, but have been incompleteness in diamond. Here we show vertical-type p-channel diamond MOSFETs with trench structures and drain current densities equivalent to those of n-channel wide bandgap devices for complementary inverters. We use two-dimensional hole gases induced by atomic layer deposited Al_2_O_3_ for the channel and drift layers, irrespective of their crystal orientations. The source and gate are on the planar surface, the drift layer is mainly on the sidewall and the drain is the p^+^ substrate. The maximum drain current density exceeds 200 mA mm^−1^ at a 12 µm source-drain distance. On/off ratios of over eight orders of magnitude are demonstrated and the drain current reaches the lower measurement limit in the off-state at room temperature using a nitrogen-doped n-type blocking layer formed using ion implantation and epitaxial growth.

## Introduction

Complementary power inverters and converters composed of high-voltage n-channel field-effect transistors (FETs), which are known as n-FETs, and p-channel FETs (p-FETs) will realize high-speed operation and high power efficiency using simple gate drive circuits. When using wide bandgap semiconductors, however, high-voltage p-FETs do not show device performance comparable to that of n-FETs. Diamond is considered to be a promising high-power p-FET material because of its high hole mobility (3800 cm^2^ V^−1^ s^−1^)^1^, high-density boron acceptor doping^2^ properties and two-dimensional hole gas (2DHG) layer^3^. The 2DHG was formed on a hydrogenated (C-H) diamond surface that was covered with negatively charged adsorbates or films^4^. The carrier density of the 2DHG layer^5,6^ was as high as ~10^13^ cm^−2^ and the maximum drain current density of 2DHG diamond MOSFETs^7,8^ has been reported to be more than 1 A mm^−1^. Passivation of the C-H diamond surface by Al_2_O_3_ atomic layer deposition (ALD) improved the reliability of 2DHG diamond FETs^9,10^ and the 2DHG layer is stable up to 500 °C when Al_2_O_3_ is used simultaneously as the gate insulator and as a passivation layer and deposited by high-temperature ALD at 450 °C^11^. Stable operation of lateral-type devices in a wide temperature range^12,13^ of 10–673 K with high breakdown voltages^13,14^ of 1500–2000 V has also been reported.

The lower specific on-resistance (given in units of Ω cm^2^) that is required for power devices is obtained using a smaller device area in a horizontal structure. A FET with a vertical structure reduces its horizontal size by vertical expansion of the large drift region at which the high voltage is applied. Vertical FETs with trench gates have been commercialized as power MOSFETs in Si and SiC^15^ and were recently developed in GaN^16^. These devices use bulk conduction. While the two-dimensional electron gas (2DEG) layer at a GaN/AlGaN interface has high electron mobility and has been used to form high-electron-mobility transistors (HEMTs), it is difficult to form a 2DEG layer in the vertical direction because the 2DEG appears only on the (0001) GaN surface. Therefore, vertical-type GaN MOSFETs use bulk conduction without using the high mobility of the 2DEG drift layer.

2DHG layers on diamond surfaces are advantageous for fabrication of vertical-type devices with trench structures. 2DHG layers can be formed on hydrogenated diamond surfaces independently of their crystal orientations. Because of the high trench coverage of the ALD process, ALD-Al_2_O_3_ is used for gate insulation and passivation layers for 2DHG diamond MOSFETs in 3D structures. Diamond lateral and vertical-type 2DHG diamond MOSFETs with trench structures^17^ and triple-gate field-effect transistors^18^ were fabricated using inductively coupled plasma reactive ion etching (ICP-RIE) to form their trench structures and fin patterns, respectively, and both devices have 3D channel regions. In this work, we fabricate vertical-type 2DHG diamond MOSFETs with trench structures on highly boron-doped (p^+^) single crystal diamond substrates and obtain device operation equivalent to that of devices with lateral structures with the same gate-drain distance.

## Results

**Figure 1:**
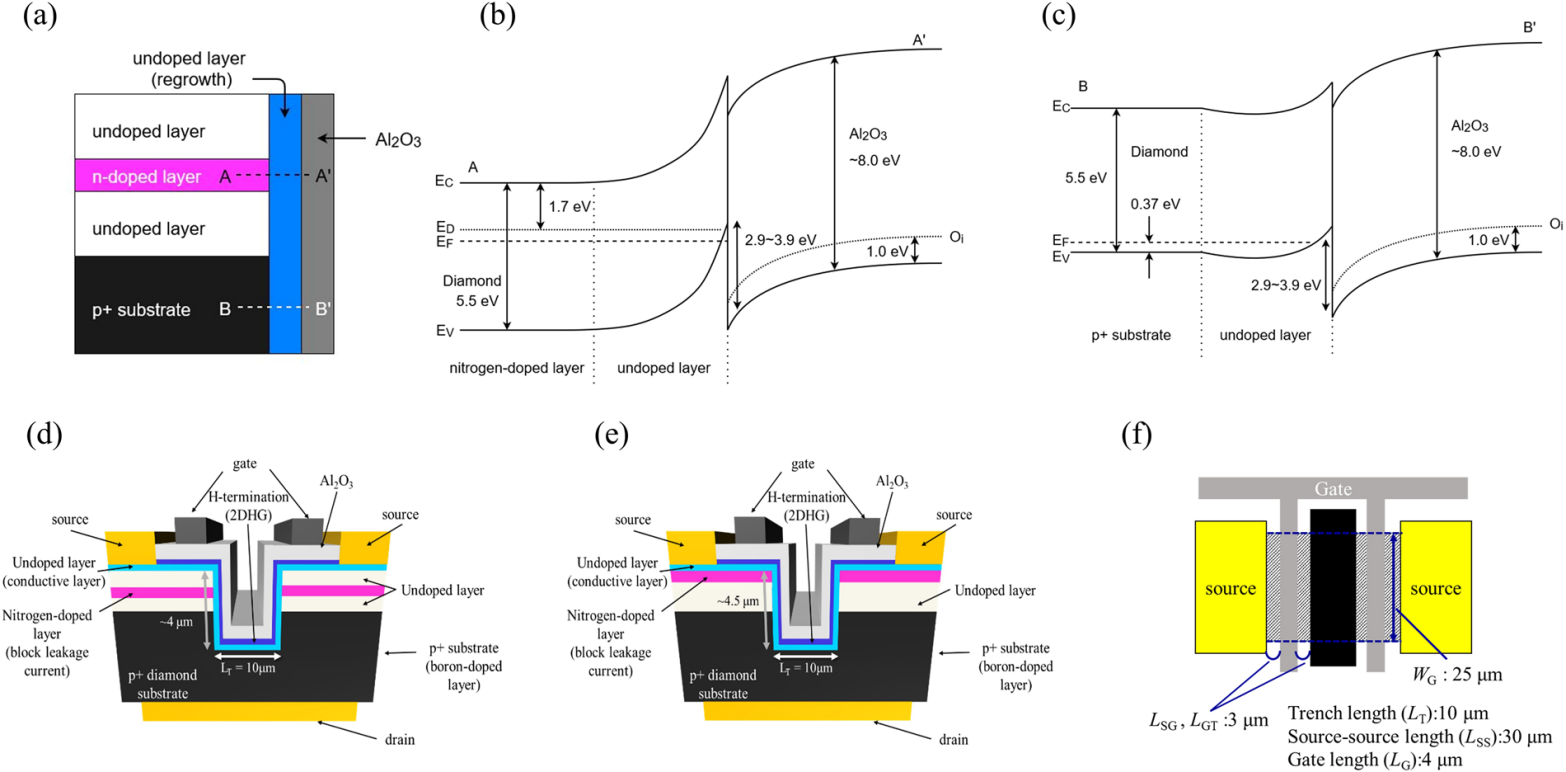
Schematic diagrams of the 2DHG diamond MOSFETs. (**a**) Schematic diagram at trench structure. (**b**) Band diagram at A-A’ in (a). (**c**) Band diagram at B-B’ in (a). (**d**) Cross-section views of vertical-type 2DHG diamond MOSFETs with N implanted layer. (**e**) With N doped epitaxial layer. (**f**) Top view.

To fabricate vertical p-channel FET structures with low hole current leakage in the vertical direction, highly resistive n-type layers are required. A nitrogen-doped layer satisfies this requirement because nitrogen is a deep donor with activation energy of 1.7 eV and keeps the Fermi level E_F_ above the intrinsic Fermi level Ei located at almost middle point of energy gap. Simple enlarged view of the trench portion is shown in Fig. 1(a) and band diagram at nitrogen-doped layer/undoped layer/Al_2_O_3_ (A-A’ in Fig. 1(a)) and p+-type substrate/undoped layer/Al_2_O_3_ (B-B’ in Fig. 1(a)) placed trench structure is shown in Fig. 1(b,c). Valence band offset is 2.9–3.9 eV^19,20^.

The device structure has a major influence on the device characteristics. Two vertical-type 2DHG diamond MOSFET structures are shown in cross-section views (Fig. 1(d,e)) and from above (Fig. 1(f)). The common aspect of these two vertical MOSFETs is the 2DHG layer that is formed on the C-H diamond surface, with the trench and the sidewall acting as the channel and drift regions. The main difference is that two different n-type blocking layers were fabricated by nitrogen hot-implantation with high-temperature post-annealing after growth (Fig. 1(d)) and nitrogen incorporation during growth (Fig. 1(e)). We used p^+^ diamond substrates synthesized by the high-pressure high-temperature (HPHT) method for vertical-type devices with N-implanted layer, as shown in Fig. 1(d), and a p^+^ conduction layer deposited by microwave plasma chemical vapour deposition (MPCVD) on an n-type diamond substrate (where the layer includes part of the back side of the substrate) for devices with N-doped epitaxial growth, as shown in Fig. 1(e). (Detail of substrate covered by boron doped layer is shown in Supplementary 1).

We then successfully formed high-concentration (~10^19^ cm^−3^) nitrogen-doped layers either by nitrogen ion implantation into pure diamond at high energy (1.7 MeV) or nitrogen incorporation during epitaxial growth using MPCVD.

The p-type conducting region was limited to the sub-surface of the homoepitaxial layer (200 nm thick), which is shown as the light blue layer in Fig. 1(d,e), and was regrown after trench formation. The gate length was fixed at 4 µm. The trench depths of the two devices in Fig. 1(d,e) were set at ~4 and ~4.5 µm, respectively. Here, we define the effective drift region as the sub-surface of the regrown layer ranging from the gate edge to the top corner of the p^+^ region near the trench sidewall, as shown in Fig. 1(d). The lengths from the gate edge to the trench entrance and from the entrance to the p^+^ top corner were 3 µm and 2 µm, respectively. Therefore, the total drift layer length between the gate and the drain was ~5 µm. The distance from the p^+^ top corner to the trench bottom is the overlap depth (~2 µm) of the 2DHG layer with the p^+^ region and is sufficient for hole conduction from the 2DHG to the p^+^ region. The 2DHG channel width (*W*_G_  in Fig. 1(f)) was fixed at 25 µm.

### Current-voltage characteristics

**Figure 2:**
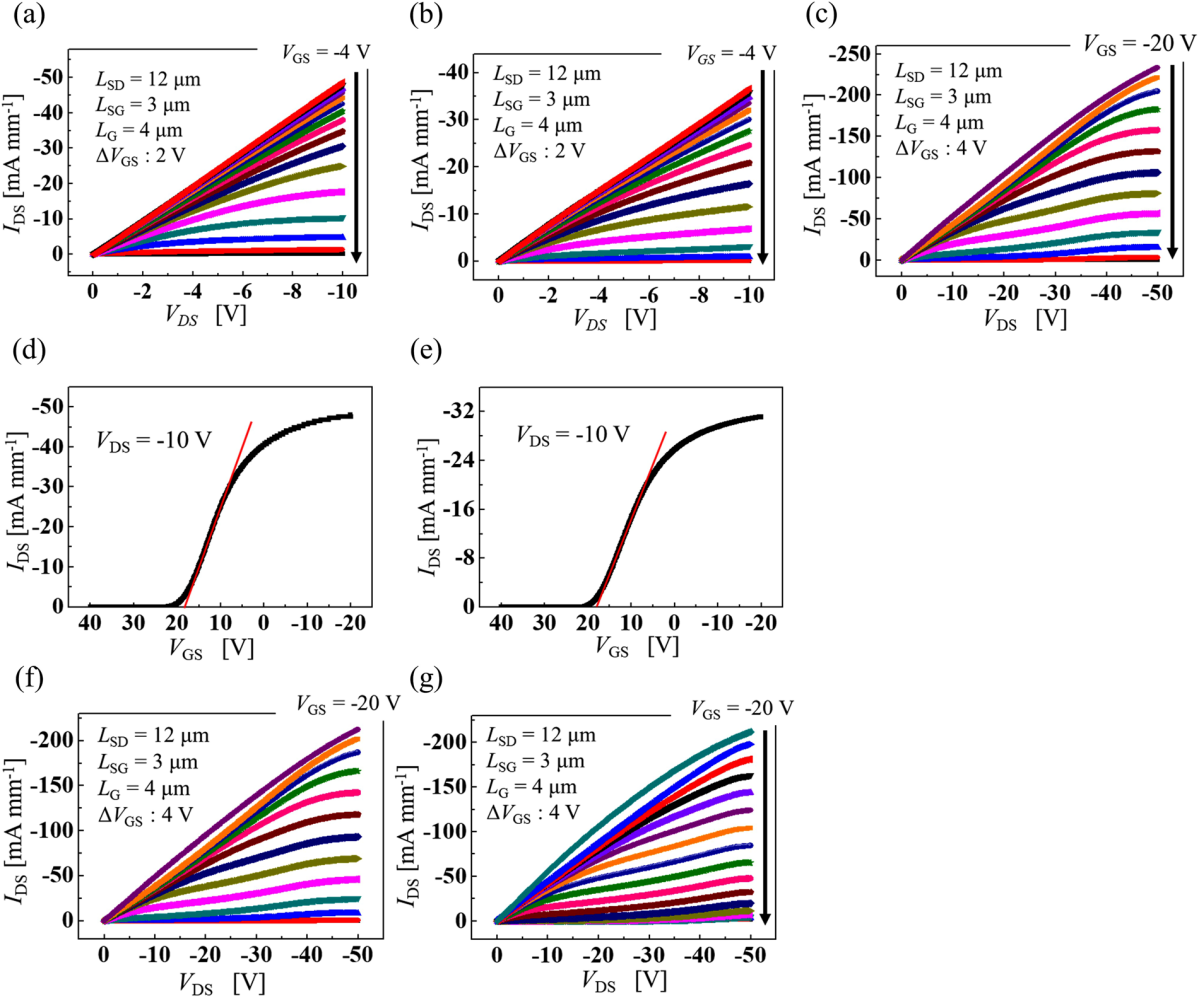
*I–V* characteristics of both kinds of vertical-type 2DHG diamond MOSFETs. *I*–*V* characteristics. (**a**) *I*_DS_–*V*_DS_ characteristics of vertical-type device with N implanted layer. *V*_DS_ ranges up to −10 V and *V*_GS_ is varied from −4 V to 26 V in steps of 2 V. (**b**) With N doped epitaxial layer. (**c**) With N implanted layer. *V*_DS_ ranges up to −50 V and *V*_GS_ is varied from −20 V to 28 V in steps of 4 V. (**d**) *I*_DS_–*V*_GS_ characteristics of vertical-type device with N implanted layer at *V*_DS_ of −10 V. (**e**) With N doped epitaxial layer. (f) *I*_DS_–*V*_DS_ characteristics of vertical-type device with N implanted layer at room temperature (RT). (**g**) At 200 °C.

Both kinds of the vertical-type 2DHG diamond MOSFETs fabricated in this study exhibited current-voltage characteristics that are comparable to those of lateral-type diamond MOSFETs. Figure 2(a,b) show two typical drain-source current (*I*_DS_) versus drain-source voltage (*V*_DS_) characteristics for the two vertical-type 2DHG diamond MOSFETs over a gate-source voltage (*V*_GS_) range from −4 to 26 V with voltage steps of 2 V. Their drain current densities at a *V*_DS_ of −10 V are 48.9 mA mm^−1^ (with N-implanted layer) and 36.7 mA mm^−1^ (with N-doped epitaxial layer), respectively. The maximum field-effect mobility and specific on-resistance values of these MOSFETs are 98 cm^2^ V^−1^ s^−1^ and 31 mΩ cm^2^ and 74 cm^2^ V^−1^ s^−1^ and 41 mΩ cm^2^, respectively. Because we only used the left side source electrode for the *I*-*V* measurements, we adopted a device horizontal area equivalent to half-length between the two source electrodes composed of the surface channel and the drift area (*L*_SG_ + *L*_G_ + *L*_GT_ + *L*_T_/2 = 15 µm in Fig. 1(f)) with the channel width (*W*_G_ = 25 µm) to calculate the specific on-resistance. The source-to-source length (*L*_SS_) will be reduced in the near future by downscaling of the trench by a factor of 10. Figure 2(c) shows further *I*_DS_ − *V*_DS_ characteristics for *V*_DS_ ranging from 0 to −50 V and *V*_GS_ ranging from −20 to 28 V with voltage steps of 4 V. The *I*_DS_ of the vertical-type device with N-implanted layer increases with rising *V*_DS_ and the measured drain current density is 234 mA mm^−1^ at a *V*_DS_ of −50 V. The maximum current density is the highest reported in vertical-type diamond MOSFETs and is comparable to that of lateral-type 2DHG diamond MOSFETs with a similar gate-drain length (*L*_GD_) and the same ALD-Al_2_O_3_ simultaneous gate insulator and passivation layer. The drain current (*I*_DS_) increases with increasing drain voltage (*V*_DS_) from 10 V up to 40 V. At *V*_DS_ values of more than 40 V, *I*_DS_ is almost saturated.

Figure 2(d,e) shows the *I*_DS_–*V*_GS_ characteristics at a *V*_DS_ of −10 V. The threshold voltage (*V*_th_) values of the two device-types were determined from the *I*_DS_–*V*_GS_ characteristics to be 23.2 V (with N-implanted layer) and 23.0 V (with N-doped epitaxial layer), respectively, and these values are close to that of a lateral-type C-H diamond MOSFET with the same gate oxide thickness. These vertical-type 2DHG diamond MOSFETs have normally-on characteristics, which is common in general 2DHG diamond MOSFETs with Al_2_O_3_ gate insulators. The 2DHG layer is induced by the negative charges in the ALD-Al_2_O_3_ film^21^ near the interface, even at a *V*_GS_ of 0 V. The drain current was controlled well using the gate bias in a manner similar to that of lateral-type 2DHG diamond MOSFETs. This also means that the 300-nm-thick nitrogen-doped epitaxial layer or 50-nm-thick nitrogen-implanted layer effectively blocked the substrate leakage current that flows directly from the surface under the source electrode towards the drain electrode in the perpendicular direction.

### Temperature characteristics

Comparison of the *I*_DS_–*V*_GS_ characteristics at various temperatures provides further insight into the device operation. Figure 2(f,g) show the device *I*_DS_–*V*_DS_ characteristics at room temperature (RT) and 200 °C, respectively. *I*_DS_–*V*_DS_ characteristics of device with N-implanted layer at 100 °C and 300 °C, and *I*_DS_–*V*_DS_ characteristics of device with N doped epitaxial layer at RT up to 300 °C are shown in Supplementary 3. The maximum current density at *V*_DS_ and *V*_GS_ values of −50 V and −20 V was 212 mA mm^−1^ at both RT and 200 °C. For operation at RT and at 200 °C, the off-state was obtained at *V*_GS_ values below 24 V and 36 V, respectively. The gate voltage required to turn the device off increases with increasing temperature, but the change in current density with increasing temperature is small.

**Figure 3:**
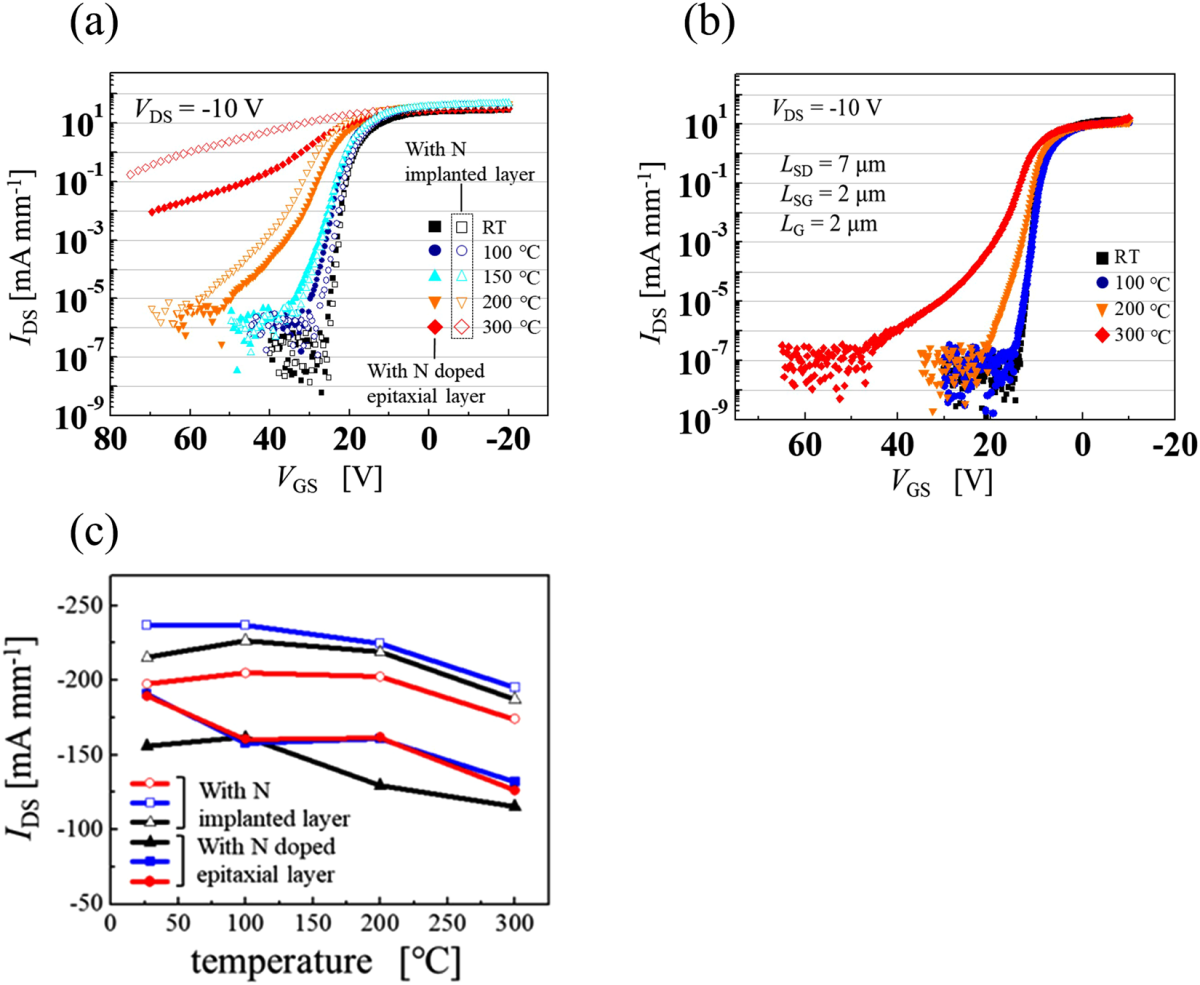
Temperature dependence of *I*_DS_–*V*_GS_ characteristics and drain current density of vertical and lateral-type devices. (**a**) *I*_DS_–*V*_GS_ characteristics at *V*_DS_ of −10 V from room temperature up to 300 °C. The characteristics of the vertical-type device with N-implanted layer are expressed using open symbols and those of devices with N-doped epitaxial layers are expressed using closed symbols. (**b**) Of lateral-type device. (**c**) Current densities of the two vertical-type devices at *V*_DS_ of −50 V and*V*_GS_ of −20 V from room temperature up to 300 °C.

This device operates with a high on/off ratio at temperatures under 200 °C. Figure 3(a) shows the logarithmic *I*_DS_–*V*_GS_ characteristics of two vertical-type 2DHG diamond MOSFETs over the range from RT to 300 °C. The characteristics of the vertical-type devices with N-implanted layer and N-doped epitaxial layers are expressed using open symbols and closed symbols, respectively. In the off-state (over 25 V), *I*_DS_ was approximately 10^−10^ A mm^−1^ for RT operation, and the on/off ratio of this device was more than eight orders of magnitude in both vertical-type devices; these values are comparable to those of lateral-type devices (shown in Fig. 3(b)). At RT and 150 °C, the drain current reached the lower measurement limit and the on/off ratios were approximately eight and seven orders of magnitude, respectively, in both devices. When compared with RT operation, the *V*_GS_ required to reach the lower limit shifted to positive values at 100 °C and 150 °C. The increase in the leakage current is remarkable at temperatures above 150 °C. At 300 °C, the drain current’s dependence on the gate voltage falls and the on/off ratios of the vertical-type devices with N-implanted and N-doped epitaxial layers were approximately one and three orders of magnitude, respectively, indicating that this device will not work properly at higher temperatures (over 300 °C). At 200 °C, the leakage current increased when compared with that at RT, but the on/off ratio was still high as approximately five orders of magnitude and the device turned off at a *V*_GS_ of 36 V (Fig. 2(g)). Figures 2(g) and 3(a) show that this device can operate properly at temperatures under 200 °C and the change in the device characteristics between RT and 150 °C is very small.

The increase in the leakage current for these vertical C-H diamond MOSFETs started at relatively low temperatures when compared with the lateral-type C-H diamond MOSFETs shown in Fig. 3(b). The lateral-type devices could operate correctly at 300 °C and still had a very high on/off ratio of approximately seven orders of magnitude (see Fig. 3(b)). This result indicates that we can even obtain the off-state of the 2DHG layer at 300 °C, and the high leakage current of the vertical-type device over the range from 200–300 °C originates from bulk conduction rather than the surface conduction layer. The small current density dependence on *V*_GS_ at 300 °C shows that the leakage current flows in the substrate in the perpendicular direction and the nitrogen-doped layer thus did not work as the leakage blocking layer at high temperatures. However, this layer could block the leakage currents at temperatures under 200 °C, despite being very thin (~50 nm).

Figure 3(c) shows the maximum current densities of six devices (three devices of each type) over the range from RT–300 °C. The current densities of the devices with the N-implanted layers at a *V*_DS_ of −50 V ranged from 197 to 235 mA mm^−1^ at RT and the variation of these values from the average value ranged from approximately 10% to 15%. This variation is caused by the surface condition of the trench sidewall, which is dependent on the plasma etching process, so the effects of the variation on both the hole mobility and the current density are significant. The maximum current density remained very stable from RT to 200 °C and the average maximum current densities for the three devices at RT and 200 °C were 220 and 217 mA mm^−1^, respectively. At 300 °C, while the average maximum current density decreased by ~15% to 187 mA mm^−1^ from that at RT, the deviation of the maximum current density at approximately 7% between devices was smaller than that at RT. This result indicates that the current flows not only through the surface 2DHG layer but also through the substrate as a leakage current. Because the current density deviation is caused by the trench surface conditions and it decreases at higher temperatures, the current must flow via another path that is not influenced by surface conditions, such as the bulk. The current densities of the vertical-type devices with N-doped epitaxial layers ranged from 156 mA mm^−1^ to 191 mA mm^−1^ at RT and from 116 mA mm^−1^ to 132 mA mm^−1^ at 300 °C. At 300 °C, the average current density decreased by ~30%. The current density decreases with increasing temperature in both vertical-type devices. The rate at which the current density decreases in the vertical-type devices with N-doped epitaxial layers is lower than that of the vertical-type devices with N-implanted layer. However, the current density remains stable over a wide temperature range when compared with that of the junction FET^22^ and that of a metal-semiconductor FET (MESFET)^23^ with a boron-doped channel. This difference in the current density change between the two vertical-type devices is caused by leakage current differences at high temperatures (i.e., over 300 °C).

### Analysis via simulations

**Figure 4:**
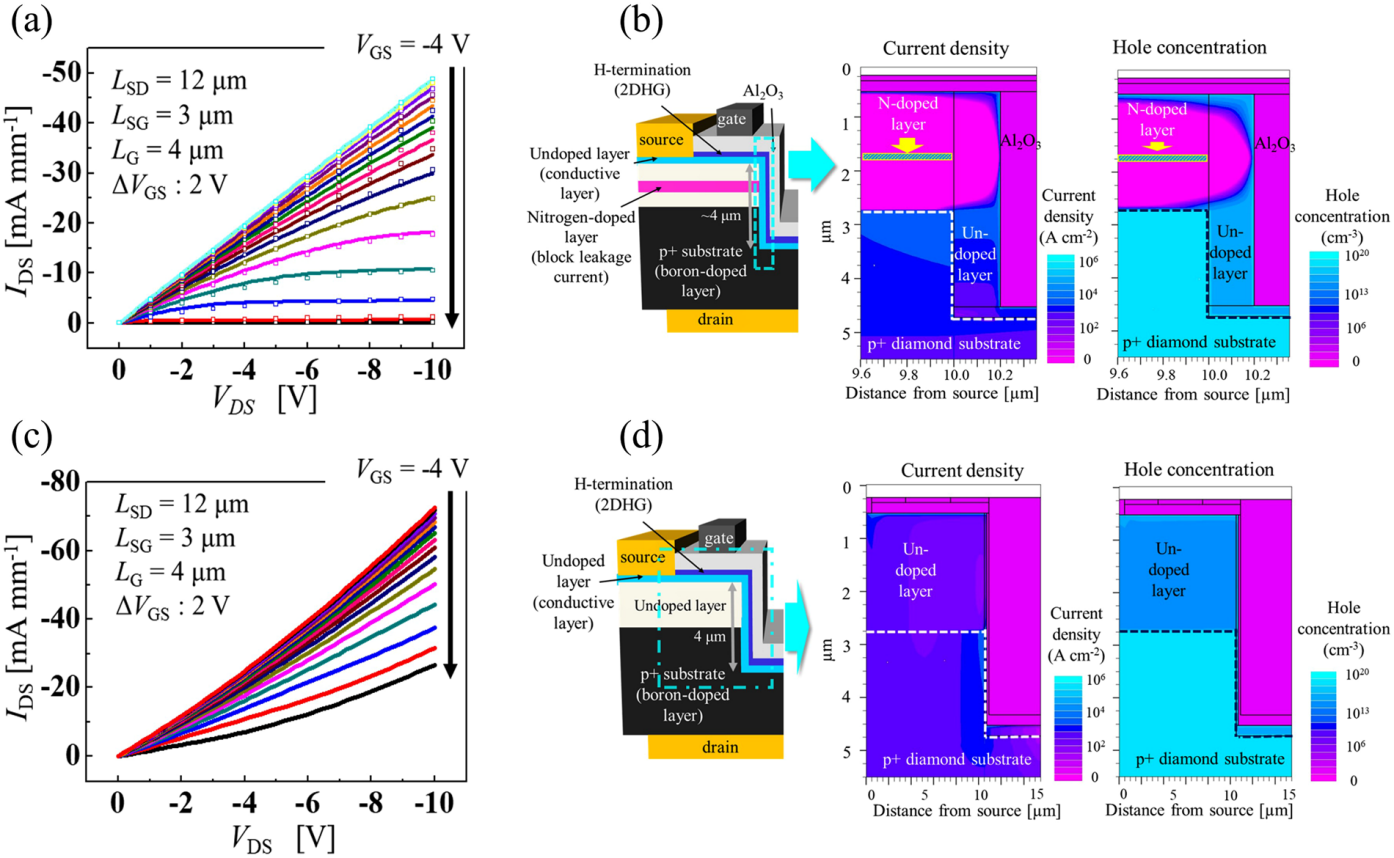
*I*_DS_–*V*_DS_ characteristics and total current densities determined by simulations based on the two-dimensional negatively charged sheet model. (**a**) *I*_DS_–*V*_DS_ characteristics of measured (plots) and simulated (solid line) results device with N implanted layer. (**b**) Total current density and hole concentration at the left side of trench (indicated by dotted line). (**c**) *I*_DS_–*V*_DS_ characteristics of vertical-type device without the nitrogen-doped blocking layer determined by simulations. (**d**) Cross-sectional view, total current density and hole concentration of vertical type device use for (**c**).

The operational simulation of the lateral 2DHG diamond MOSFETs has been performed based on the two-dimensional negatively charged sheet model upon which the 2DHG was realized and the measured *I*_DS_–*V*_DS_ curve was reproduced well^13^. The charge sheet model is often used to simulate AlGaN/GaN FETs with 2DEG for channels and can be adapted to simulation of the device characteristics of 2DHG diamond MOSFETs. For the vertical-type devices under consideration here, the main difference is the hole transport that occurs from the 2DHG drift region to the p^+^ drain region. Figure 4(a) shows the *I*_DS_–*V*_DS_ characteristics from both the measurement results and the simulated results for *V*_DS_ ranging from 0 V to 10 V. In Fig. 4(a), the measured *I*_DS_–*V*_DS_ characteristics are shown as open plots and the simulation results are shown as solid lines. It is proposed that the surface conditions inside the trench, such as those of the trench sidewall and bottom, are not the same as those of the surface outside the trench, so we varied the hole carrier mobility in each region. The optimal negative charge areal density is fixed at −6.7 × 10^12^ cm^−2^ and the hole channel mobility based on low-field mobility model in the lateral channel and the vertical channel in the trench structure are 95 cm^2^ V^−1^ s^−1^ and 43 cm^2^ V^−1^ s^−1^, respectively. The measured *I*_D_–*V*_DS_ characteristics were reproduced well when the mobility in the vertical channel in the trench structure was reduced when compared with that of the lateral channel region. These results show that the carrier mobility of the vertical channel was lower than that of the lateral channel as a result of the plasma etching, but that was still a sufficient level for the channel to act as a 2DHG layer. The damage caused by the trench fabrication process using ICP-RIE was covered by regrowth of a 200-nm-thick undoped layer.

The current flow in the vertical-type 2DHG diamond MOSFETs was also simulated. Figure 4(b) shows a cross-section view of the vertical-type device along with the total current density and the hole concentration on the left side of the trench (indicated by the dotted line) at a *V*_DS_ of −10 V and a *V*_GS_ of −4 V in the simulations. The broken line on the right side of the figure indicates the boundary between the p^+^-type diamond substrate and the undoped layer. The left and the bottom side of the broken line indicate the p^+^-type diamond substrate. The square boxed part that is laterally located in the undoped layer corresponds to the region in which nitrogen ions were implanted at high temperature with an acceleration energy of 1.7 MeV. The trench structure of this device entered the p^+^-type diamond substrate to a depth of approximately 2 µm and the current flows in the 200-nm-thick regrown undoped layer on the p^+^-type substrate. Holes are induced in the 200-nm-thick regrown undoped layer on the p+ substrate and the current flow from the 2DHG layer to the p^+^ region is higher in the upper part than at the bottom of the trench. Figure 4(b) shows that the trench structure fabricated in the p^+^-type diamond substrate is affected by the improvement in the current density and the current can flow through the thin nominally undoped layer, which is thick enough to form a 2DHG layer. In the layer, nitrogen density is low (<10^16^ cm^−3^), but exceeds boron density (~10^14^ cm^−3^). Thus, the obtained 2DHG is an inversion layer. From Fig. 4(b), current does not flow in most of the undoped layer that was deposited on the p^+^-type diamond substrate without the regrowth layer and the 2DHG layer. Results of simulation of the current density indicate that the trench bottom length does not influence the drain current density, which is low, and we can downscale the trench length to be as short as 1 µm without reducing the drain current density. Device simulation of vertical-type device with N doped epitaxial layer and without overlapping region are shown in Supplementary 4.

To confirm the importance of the action of the nitrogen-implanted layer as a leakage blocking layer, the *I*_DS_–*V*_DS_ characteristics of a vertical-type device without the nitrogen-doped layer were simulated as shown in Fig. 4(c). While the maximum current density is 72.6 mA mm^−1^ at a *V*_DS_ of −10 V and a *V*_GS_ of −4 V, this value is approximately 50% higher than that of the device with the nitrogen-doped layer produced by ion implantation; however, the device without the nitrogen-implanted layer does not turn off, even at a *V*_GS_ of 26 V, which is a sufficient gate voltage level to turn off the devices with the nitrogen-doped blocking layers. The left side of Fig. 4(d) shows a cross-section view of the vertical-type device without the nitrogen-doped layer that was used for the simulation. The total current density and the hole concentration for this type of device (surrounded by the dotted line) are shown on the right side of Fig. 4(d). The current flows directly from the source electrode to the drain through the undoped layer in the direction perpendicular to the substrate. These two patterns in the simulation results show that the nitrogen-doped n-type layer plays a significant role in blocking leakage currents.

### Breakdown characteristics

**Figure 5:**
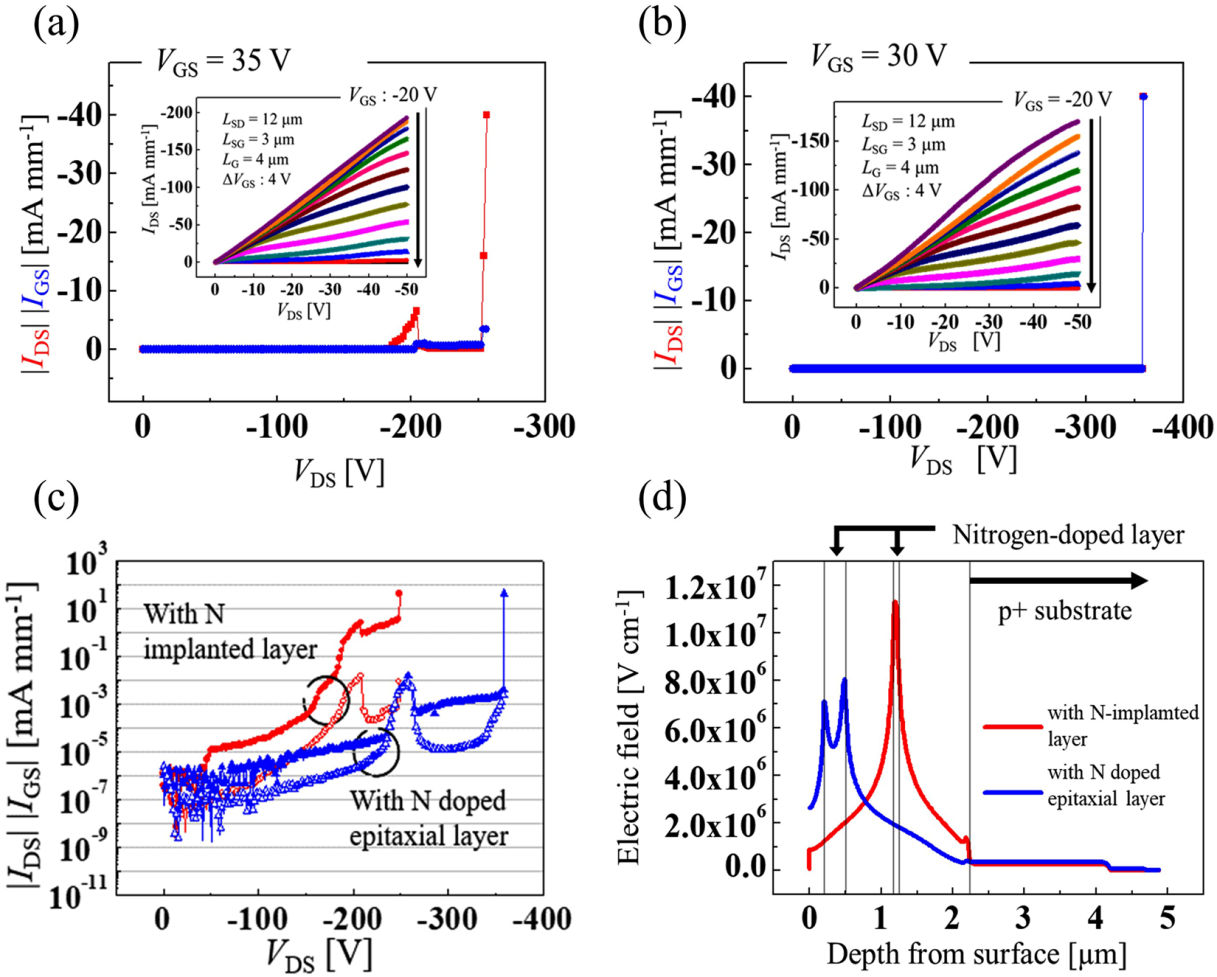
Breakdown characteristics and electric field distribution by simulation of vertical-type 2DHG diamond MOSFETs. (**a**) Breakdown characteristics and *I*_DS_–*V*_DS_ characteristics of vertical-type 2DHG diamond MOSFETs with N implanted layer. The breakdown voltage (*V*_B_) is 249 V and the drain current density (*I*_DS_) is 193 mA mm^−1^. (**b**) With N-doped epitaxial layer. *V*_B_ is 359 V and *I*_DS_ is 170 mA mm^−1^. (**c**) Breakdown characteristics of both kinds of vertical-type device on a semilogarithmic scale. *I*_DS_ and *I*_GS_ are shown using closed symbols and open symbols, respectively. (**d**) Electric field distributions of the two vertical-type devices at the undoped and nitrogen-doped layer/regrown undoped layer interface along the trench sidewall by simulation.

Figure 5(a,b) show the breakdown characteristics and the *I*_DS_–*V*_DS_ characteristics of both kinds of vertical-type C-H diamond MOSFETs. The breakdown voltage (*V*_B_) of the device with N-implanted layer, which was determined from Fig. 5(a), was 249 V and the drain current density was 193 mA mm^−1^ at a *V*_DS_ of −50 V. The breakdown characteristics were measured at a *V*_GS_ of 35 V, which is a sufficient voltage to turn this device off. The breakdown voltage (*V*_B_) of the device with the N-doped epitaxial layers as determined from Fig. 5(b) was 359 V and the drain current density was 170 mA mm^−1^ at a *V*_DS_ of −50 V. This breakdown voltage is more than 100 V higher than that of the previous device with the same effective drift region (~5 μm).

Figure 5(c) shows the breakdown characteristics of both vertical-type devices on a semilogarithmic scale. In the figure, the drain current (*I*_DS_) is shown using closed symbols and the gate current (*I*_GS_) is shown using open symbols for both device types. In both devices, the *I*_DS_ values are as low as the lower limit of the measured values at a *V*_DS_ of −50 V, but the current increases at *V*_DS_ of more than −50 V and this increase becomes noticeable when *V*_DS_ exceeds −150 V in the device with N-implanted layer. In contrast, in the device with the N-doped epitaxial layer, the drain current density is maintained at a value close to the lower measurement limit at a *V*_DS_ of less than −200 V, which is close to the breakdown voltage of the ion-implanted device. The leakage current increases with increasing drain voltage, but the rate at which it increases is lower than that in the device with N-implanted layer. These results show that the thicker nitrogen-doped layer reduces the leakage current in the high-drain-voltage region.

The total effective drift layer length was ~5 µm in both type devices. The average electric field strengths that were calculated using these values were 0.5 and 0.7 MV cm^−1^ for the both type devices and these values are lower than that of lateral-type C-H diamond MOSFETs^13^. The reduced breakdown voltage is caused by the individual structure of the vertical type C-H diamond MOSFETs. Figure 5(d) shows the electric field strengths of the device with N-implanted layer and the device with the N-doped epitaxial layer at the undoped layer and at the thin nitrogen-doped layer/regrown undoped layer interface at *V*_DS_ of −250 V and *V*_GS_ of 26 V. Results of electric field simulation of both kinds of devices are shown in Supplementary 5. In the device with N-implanted layer, the peak of electric field strength is located 1.2 µm from the surface at the 50-nm-thick nitrogen-doped layer/regrown undoped layer interface and the localized electric field strength is higher than 10 MV cm^−1^ at *V*_DS_ of −250 V. In the device with the N-doped epitaxial layer, the peak of electric field is also located at the nitrogen-doped layer/regrown undoped layer interface. However, the localized electric field is ~8 MV cm^−1^, which is lower than that of the former device with thin nitrogen-doped layer. The difference in the electric field and the breakdown voltage caused by the thickness and placement of the nitrogen-doped layer is consistent. A thinner nitrogen-doped layer causes a high electric field concentration and a 2DHG layer between the gate edge and the trench, and the trench sidewall does not play a role as a drift layer. In lateral-type devices, the peak electric field is located near the gate edge, but in vertical-type devices, the highest electric field spot is not at the gate edge but at the nitrogen-doped layer/regrown undoped layer interface. The nitrogen-doped layer is required to block the leakage current that flows in the direction perpendicular to the diamond substrate and the electric field distribution is dependent on the condition of the nitrogen-doped layer. Therefore, the nitrogen-doped layer is very important not only in blocking the leakage current but also in terms of the device breakdown characteristics.

**Figure 6:**
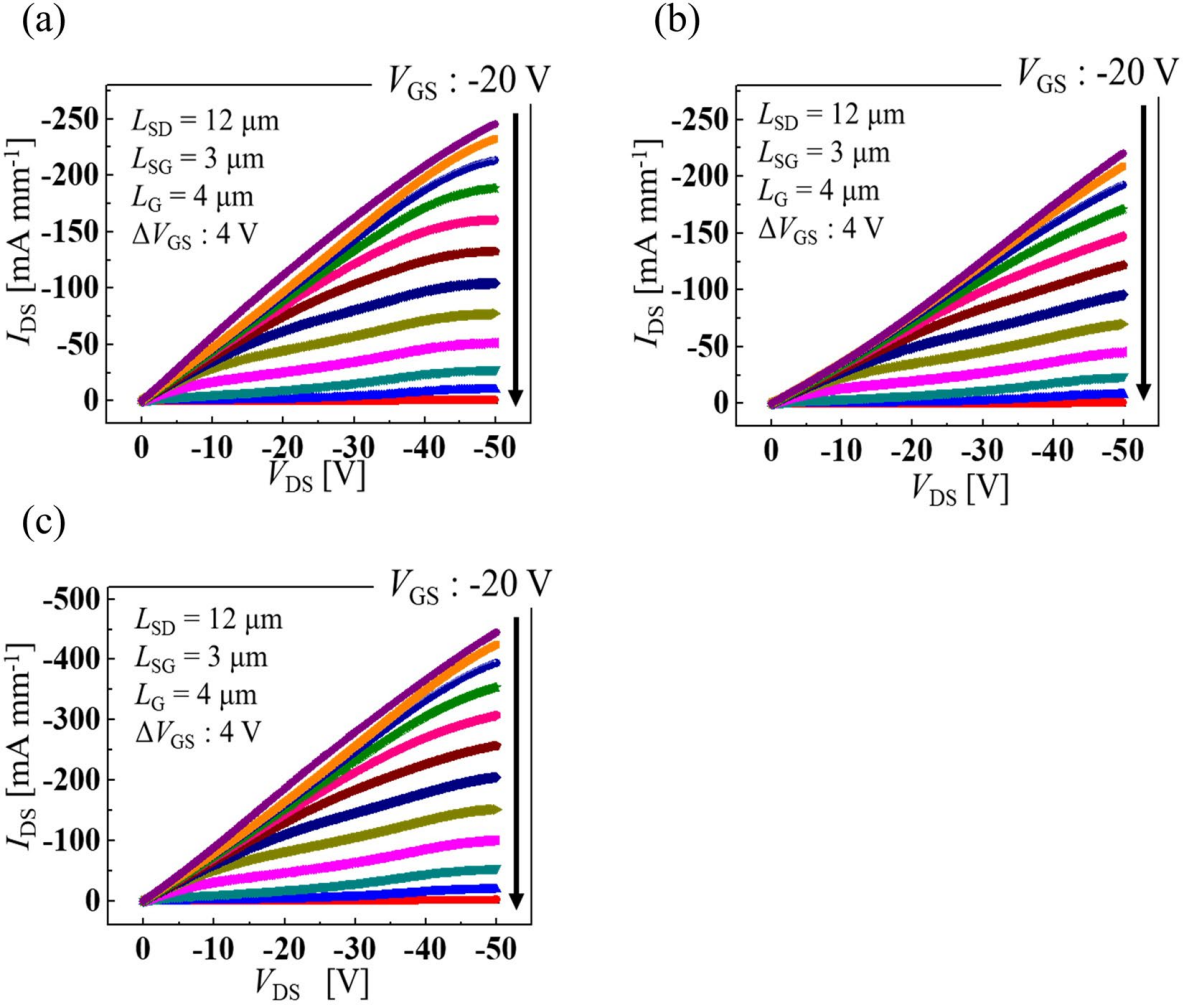
Comparison of *I*_DS_–*V*_DS_ characteristics of vertical-type 2DHG diamond MOSFETs with N implanted layer using either or both side source electrode. (**a**) *I*_DS_–*V*_DS_ characteristics of vertical-type device with N implanted layer when using left side source electrode. (**b**) Using right side source electrode. (**c**) Using both sides source electrodes.

There are two source electrodes located on both sides of the trench structure in the fabricated vertical-type C-H diamond MOSFETs. Figure 6(a,b,c) show the *I*_DS_–*V*_DS_ characteristics of vertical-type C-H diamond MOSFETs with N-implanted layer when using only the left-side electrode, only the right-side electrode and using both side electrodes, respectively. The drain current density is normalized by channel width (*W*_G_) of 25 µm. The maximum drain current density of the vertical-type device is 245 mA mm^−1^ when using only the left-side electrode and 220 mA mm^−1^ when using only the right-side electrode. The difference in the drain current density is caused by the condition of the trench sidewall and the slight displacement of the source electrode position with respect to the trench structure. The maximum drain current density of the vertical-type device when both side source electrodes are used is 444 mA mm^−1^. In this case, the maximum current density is slightly lower than the total current density when using either side source electrode alone. The overlapping of the channel region at the trench bottom causes the reduction in the current density. However, the drain current of the vertical-type device when using both side electrodes is close to the total drain current when using the left- or right-side source electrode only, because the drain current mainly flows through the regrown undoped layer at the trench sidewall. The sidewall length for both vertical-type C-H diamond MOSFETs is 10 µm and the current density when standardized with respect to the channel area is ~1500 A cm^−2^ and this value is higher than diamond junction FET (JFET)^20^. *I*_DS_–*V*_DS_ characteristics standardized by channel area is shown in Supplementary 6. In the vertical-type C-H diamond MOSFET presented here, both the wide trench structure and the drift region at the substrate surface are unnecessary because all the drift current passes through the sidewall and not the bottom of the trench, as shown in Fig. 4(b). The horizontal area of the device can be reduced to approximately one quarter of the existing size by fabricating the gate electrode in the trench structure and reducing the sidewall distance, and the standardized current density will be more than 5000 A cm^−2^.

## Summary

We have fabricated vertical-type 2DHG diamond MOSFETs with nitrogen-doped blocking layers formed by either ion implantation or epitaxial growth with maximum current densities (234 mA mm^−1^ and 191 mA mm^−1^, respectively, at *V*_DS_ of −50 V and *V*_GS_ of −20 V) and an on/off ratio (~10^8^) that were comparable to those of lateral-type devices at room temperature. In the trench structure, the current flows in the 2DHG layer of the thin undoped layer and the p^+^-type diamond substrate. From the *I*_DS_–*V*_DS_ characteristics of these two device types, the specific on-resistance of the vertical-type device is comparable to that of a lateral-type device. The results reported here therefore show that the condition of the trench sidewall surface is close to that of the substrate surface and the approximately 200-nm-thick undoped layer is not highly resistive. The *I*_DS_–*V*_GS_ characteristics and the on/off ratio show that current flows in the 2DHG layer and the nitrogen-doped layer blocks the substrate leakage current well.

## Methods

In this report, we used a p^+^-type HPHT (001) single crystalline diamond substrate to fabricate vertical-type hydrogen-terminated diamond MOSFETs with N-implanted layer. For the vertical device with the N-doped epitaxial layer, we used n-type diamond covered with a boron-doped layer. The boron concentration of the p^+^-type diamond was 10^19^–10^20^ cm^−3^, so we regard this substrate as being part of the drain. Initially, for device with N-implanted layer, we deposited a 2.0-µm-thick undoped epitaxial layer to develop a drift layer by microwave plasma chemical vapor deposition (MPCVD) on the p^+^-type diamond substrate. The growth temperature, time and chamber pressure for the undoped epitaxial layer were 600 °C, 15 h 20 min and 35 Torr, respectively. The H_2_, CH_4_ and CO_2_ flow rates were 394, 3 and 3 sccm, respectively. An additional n-type diamond layer was fabricated by 1.7 MeV hot implantation of nitrogen ions. The temperature during implantation and fluence were 800 °C and 2 × 10^14^ cm^−2^, respectively. From Stopping and Range of Ions in Matter (SRIM) simulations, a nitrogen-doped n-type diamond layer was fabricated at a depth of approximately 1 µm from the substrate surface and the nitrogen concentration was ~10^19^ cm^−3^. The nitrogen-ion implanted layer thick was approximately 50 nm. (doping profile by SRIM is shown in Supplementary 2) After nitrogen ion implantation, the diamond substrate was annealed at 1500 °C for 2 h. The chamber pressure was 1 atm and the atmosphere was Ar. For device with N-doped epitaxial layer, we deposited undoped and nitrogen-doped epitaxial layer on n-type substrate covered by boron-doped layer. The growth temperature, time and chamber pressure for the undoped epitaxial layer were 600 °C, 13 h and 35 Torr, respectively. The H_2_, CH_4_ and CO_2_ flow rates were 394, 3 and 3 sccm, respectively. The growth temperature, time and chamber pressure for the nitrogen-doped epitaxial layer were 600 °C, 6 h and 35 Torr, respectively. The H_2_, CH_4_, CO_2_ and N_2_ flow rates were 393, 3, 3 and 1 sccm, respectively.

To fabricate the trench structure, the PMGI-SF5/TSMR-V90-CP27 positive photoresist bilayers were coated sequentially on the diamond substrate using a spin coater and exposed using a photolithography system. The baking temperatures and times for the PMGI-SF5 and TSMR-V90-CP27 layers were 180 °C and 5 min and 90 °C and 1.5 min, respectively. MgO was deposited using a dual ion beam sputtering system (DIBS) as a mask for the ICP-RIE process. The MgO layer thickness was 150 nm. The trench structure was fabricated by ICP-RIE using O_2_ gas plasma. The gas flow, etching power and gas pressure were 90 sccm, 400 W and 0.5 Pa, respectively. The trench depth and length (*L*_T_) were 4 µm and 10 µm, respectively. The MgO was removed using a mixed acid solution (HNO_3_ and H_2_SO_4_ with a volume ratio of 1:3).

An additional undoped layer was deposited by MPCVD to cover the entire surface, including the trench structure, and form the 2DHG channel. The growth temperature and the chamber pressure were the same as the values given above. The H_2_, CH_4_ and CO_2_ flow rates were 400, 0.4 and 0.4 sccm, respectively. The growth time was 5 h and the undoped layer thickness was 200 nm. Ti/Au structures were deposited on the diamond substrate on both sides of the trench to act as source electrodes by electron beam (EB) evaporation. The thicknesses of the Ti and Au layers were 30 nm and 100 nm, respectively. The source electrode pattern was formed by photolithography and we used the TSMR-V90-CP27 positive photoresist. To form TiC, the sample was annealed at 500 °C for 30 min. To induce the 2DHG layer, hydrogen termination was performed over the whole substrate surface using remote hydrogen plasma. The plasma power, plasma temperature, chamber pressure and distance between sample and probe were 70 W, 620 °C, 20 Torr, and 50 mm, respectively. The H_2_ gas flow was 100 sccm. The substrate surface without the channel region but including the trench structure was oxygen-terminated using an oxygen plasma treatment for isolation. The plasma power and treatment time were 300 W and 2 min, respectively. We used the TSMR-V90-CP27 positive type resist as a mask for isolation. A 200-nm-thick Al_2_O_3_ layer was deposited by ALD to act as a gate insulation and passivation layer for hydrogen termination at 450 °C where the oxidant was H_2_O. To expose the source electrodes, the substrate was coated with a resist layer along with the isolation process mask and the 200-nm-thick Al_2_O_3_ layers without resist coating were wet-etched using a 2.38% tetramethylammonium hydroxide (TMAH) solution. Ti/Au layers were deposited on the back side of the diamond substrate to act as drain electrodes by Ion Beam Sputtering system. The thicknesses of the Ti and Au layers were 10 nm and 250 nm, respectively. The gate electrode pattern was fabricated by photolithography under the same conditions that were used to form the MgO mask pattern. Al was deposited using a thermal evaporation system and the Al layer thickness was 100 nm. In this work, the gate electrodes were formed between the trench structure and the two sides of the source electrodes.

## Electronic supplementary material

Supplementary information
