# Supplementary material for: Vertical-type two-dimensional hole gas diamond metal oxide semiconductor field-effect transistors
Source: Sci Rep. 2018 Jul 13;8:10660. doi: 10.1038/s41598-018-28837-5 (PMC6045668; doi:10.1038/s41598-018-28837-5)
Supplement: Supplementary file 1 — Supplementary information [file 41598_2018_28837_MOESM1_ESM.docx]

Supplementary information

Title: Vertical-type two-dimensional hole gas diamond metal oxide semiconductor field-effect transistors

Author

Nobutaka Oi, Masafumi Inaba, Satoshi Okubo, Ikuto Tsuyuzaki, Taisuke Kageura, Shinobu Onoda, Atsushi Hiraiwa, Hiroshi Kawarada*

Faculty of Science and Engineering, Waseda University, 3-4-1, Ohkubo, Shinjuku-ku, Tokyo 169-8555, Japan

Nobutaka Oi, Satoshi Okubo, Ikuto Tsuyuzaki, Taisuke Kageura, Masafumi Inaba & Hiroshi Kawarada*

Research Organization for Nano & Life Innovation, Waseda University, 513 Waseda-tsurumaki, Shinjuku-ku, Tokyo 162-0041, Japan

Masafumi Inaba, Atsushi Hiraiwa & Hiroshi Kawarada*

Institute of Materials and Systems for Sustainability, Nagoya University, Furo-cho, Chikusa-ku, Nagoya 464-8603, Japan

Masafumi Inaba & Hiroshi Kawarada*

National Institutes for Quantum and Radiological Science and Technology, 1233 Watanuki-cho, Takasaki-shi, Gunma 370-1292, Japan

Shinobu Onoda.

Institute of Materials and Systems for Sustainability (Tokyo Branch), Nagoya University, Bldg. 120-5 (Waseda University), 513 Waseda-tsurumaki, Shinjuku-ku, Tokyo 162-0041, Japan

Atsushi Hiraiwa & Hiroshi Kawarada*

The Kagami Memorial Laboratory for Materials Science and Technology, Waseda University, 2-8-26 Nishiwaseda, Shinjuku-ku, Tokyo 169-0051, Japan

Hiroshi Kawarada*

*Tel: +81-3-5286-3391; E-mail: kawarada@waseda.jp

Supplementary 1: substrate use for vertical-type device (Pseudo IIb substrate).

We used semi insulating n-type diamond substrate formed byhigh temperature high pressure (HPHT) for vertical-type device. For quasi-vertical conduction, boron doped p+-type layer was deposited by microwave plasma chemical vapor deposition (MPCVD). We used dedicated stage to deposit boron doped layer on the back side for vertical conduction. This stage has cross groove to deposited boron-doped layer on a part of back side substrate. The groove depth and width are 1.5 and 1 mm.

The temperature during growth, time and chamber pressure for boron-doped epitaxial layer were 700 °C, 4.5 h and 10 kPa, respectively. The H_2_, CH_4_ and trimethylboron (TMB) flow rates were 193, 2 and 5 sccm, respectively and TMB was 1 percent dilution by H­_2_. The boron concentration and the thickness were ~10^20^ cm^-3^ and ~3 µm, respectively. The schematic diagram of pseudo IIb substrate is shown in Figure. S1.


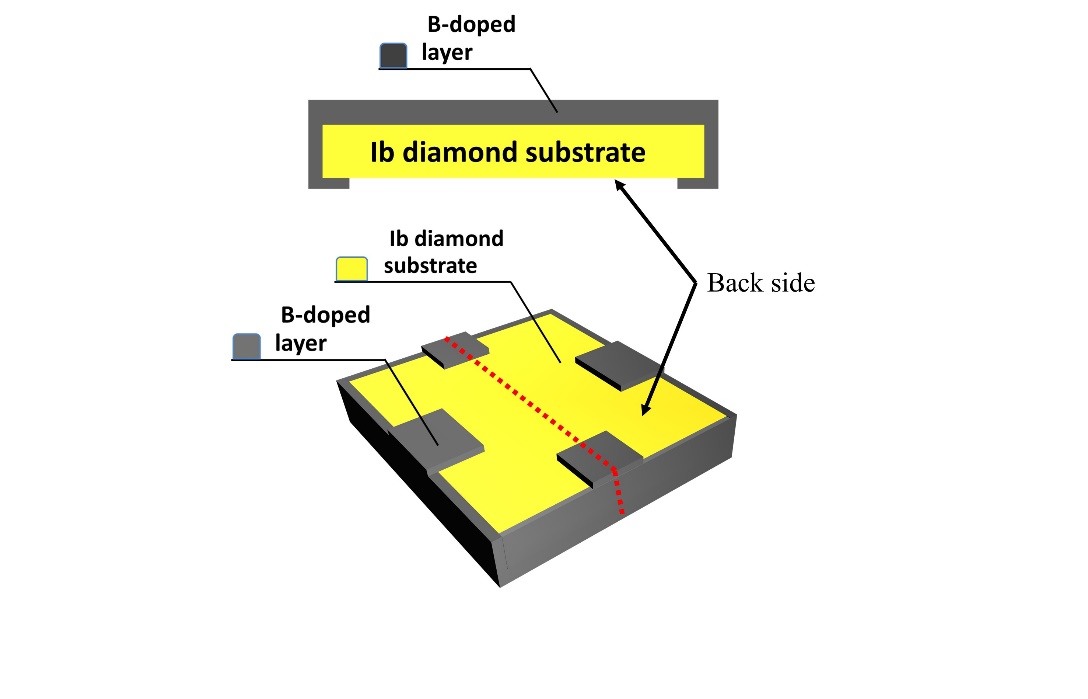


Figure. S1 Cross-sectional image of pseudo IIb substrate of the dotted line portion in the figure below and schematic view seen from back side of substrate.

Supplementary 2: Ion implantation condition.

The nitrogen concentration profile by The Stopping and Range of Ions in Matter (SRIM) is shown in Figure. S2. The energy, temperature during implantation and fluence were 1.7 MeV, 800°C and 2×10^14^ cm^−2^, respectively. The maximum nitrogen concentration was ~10^19^ cm^-3^ at 1 µm depth from surface. And the thickness was about 50 nm.


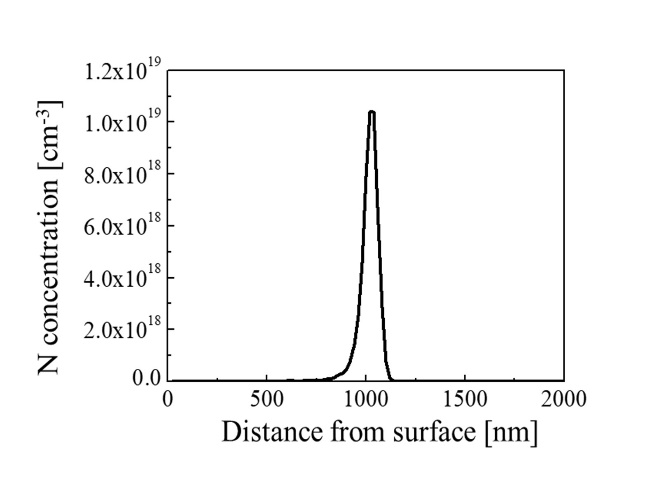


Figure. S2 Nitrogen-ion implantation profile.

Supplementary 3: *I*_DS_-*V*_DS_ characteristics of both kinds of vertical device at room temperature (RT) up to 300 ℃.

Figure. S3(a)(b) show *I*_DS_-*V*_DS_ characteristics of vertical-type device with N implanted layer at 100 ℃ and 300 ℃. *I*_DS_-*V*_DS_ characteristics at RT and 200 ℃ are shown in Figure. 2(f)(g), respectively. Figure. S3(c)~(f) shows *I*_DS_-*V*_DS_ characteristics of vertical-type device with N doped epitaxial layer at RT up to 300 ℃.

Measured drain current density of vertical-type device with N implanted layer at 100 ℃ and 300 ℃ are 219 and 182 mA mm^-1^ at *V*_DS_ of -50 V and *V*_GS_ of -20 V and with N doped epitaxial layer at RT, 100 ℃, 200 ℃ and 300 ℃ are 190, 158, 161 and 132 mA mm^-1^ at *V*_DS_ of -50 V and *V*_GS_ of -20 V.

In both kinds of vertical-type device, devices don’t turn off at 300 ℃. But in low drain voltage region (*V*_DS_), leakage current of device with N doped epitaxial layer is lower than that of device with N implanted layer. This result consistent with *I*_DS_-*V*_GS_ characteristics shown in Figure. 3(a).


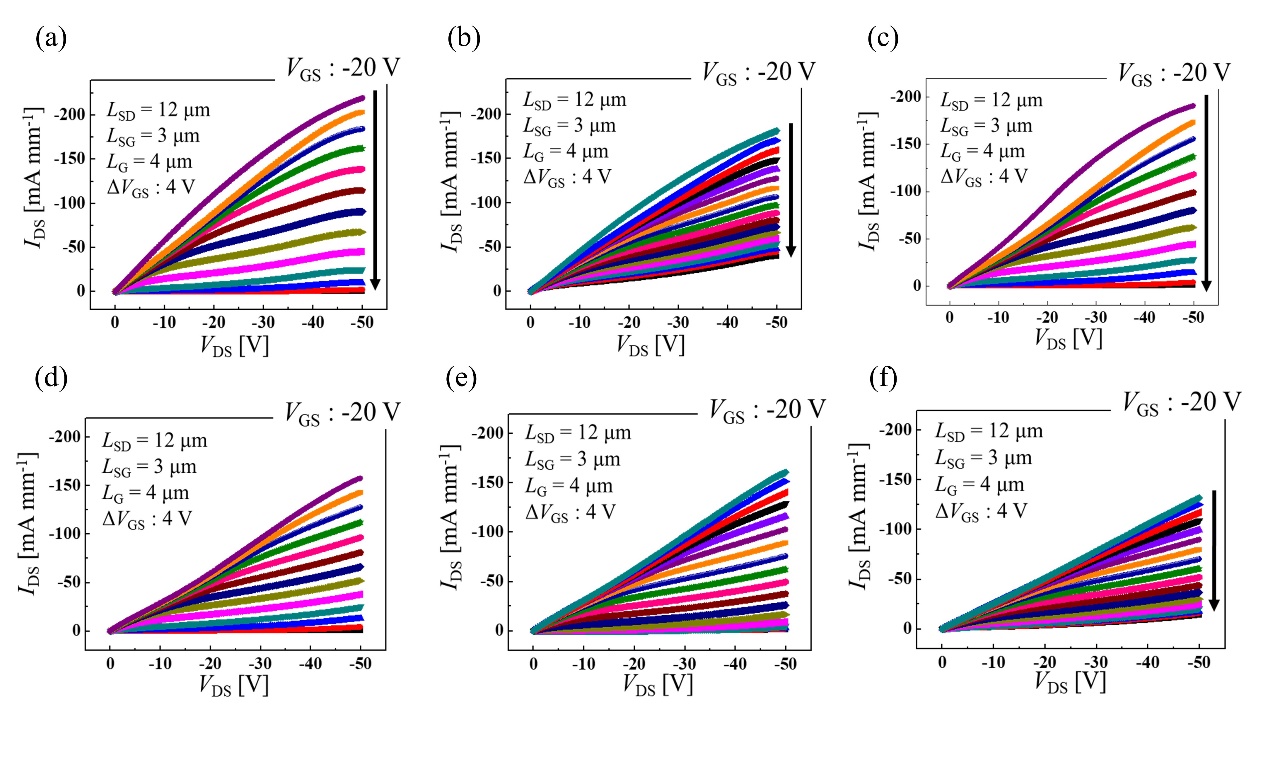


Figure. S3 *I*_DS_-*V*_DS_ characteristics of vertical-type device at RT up to 300 ℃

(a) *I*_DS_-*V*_DS_ characteristics of vertical-type device with N implanted layer at 100 ℃. (b) 300 ℃. (c) *I*_DS_-*V*_DS_ characteristics of vertical-type device with N doped epitaxial layer at RT. (d) 100 ℃. (e) 200 ℃. (f) 300 ℃.

Supplementary 4: Simulation results of vertical type device with N doped epitaxial layer.

Figure. S4(a) shows the *I*_DS_–*V*_DS_ characteristics from both the measurement results and the simulated results of vertical type device with N doped epitaxial layer. In Figure. S4(a), the measured *I*_DS_–*V*_DS_ characteristics are shown as open plots and the simulation results are shown as solid lines. The optimal negative charge areal density is fixed at −6.4×10^12^ cm^-2^ and the hole channel mobilities in the lateral channel and the vertical channel in the trench structure are 81 cm^2^ V^-1^ s^-1^ and 43 cm^2^ V^-1^ s^-1^, respectively. By using this simulation model, we succeeded in reproducing device characteristics in both kind of vertical-type 2DHG diamond MOSFTEs sufficiently.

Figure. S4(b) shows a cross-section view of the vertical-type device along with the total current density and the hole concentration on the left side of the trench (indicated by the dotted line) at a *V*_DS_ of −10 V and a *V*_GS_ of −4 V in the simulations. This simulation results suggest that leakage current is blocked by nitrogen-doped layer as well as shown in Figure. 4(b). Hole distribution and current flow are slightly different from device with N implanted, which produce difference of current density between both kinds of vertical-type device.

The relationship between the trench depth and the highest p+ diamond level was also analyzed via simulations. In our device, the trench structure overlapped with the p+-type diamond substrate by approximately 2 µm. Figure S4(c) and (d) show the *I*_DS_–*V*_DS_ characteristics and cross-section view of a vertical-type device with a trench that does not dig into the p+-type diamond substrate. In this device, the trench bottom is almost at the same level as the interface between the undoped layer and the p+-type diamond substrate. Therefore, there is a 200-nm-thick regrown undoped layer between the 2DHG layer and the p+-type diamond substrate. The drain current is substantially the same level as that of Fig.4(a). All other conditions for the simulation are the same as the conditions given above. From Figure. S4(c), the maximum current density at a *V*_DS_ of -10 V is −42.9 mA mm^−1^. This current density is slightly lower than that in the trench with the overlapping p+ layer. The reduction in the maximum current density between these two types of structure is approximately 10 % and this difference will be greatly enhanced when the trench bottom does not reach the p+ layer; the overlapping p+ layer near the bottom of trench is beneficial for effective electrical contact at the drain region while allowing for the effects of the fabrication process.


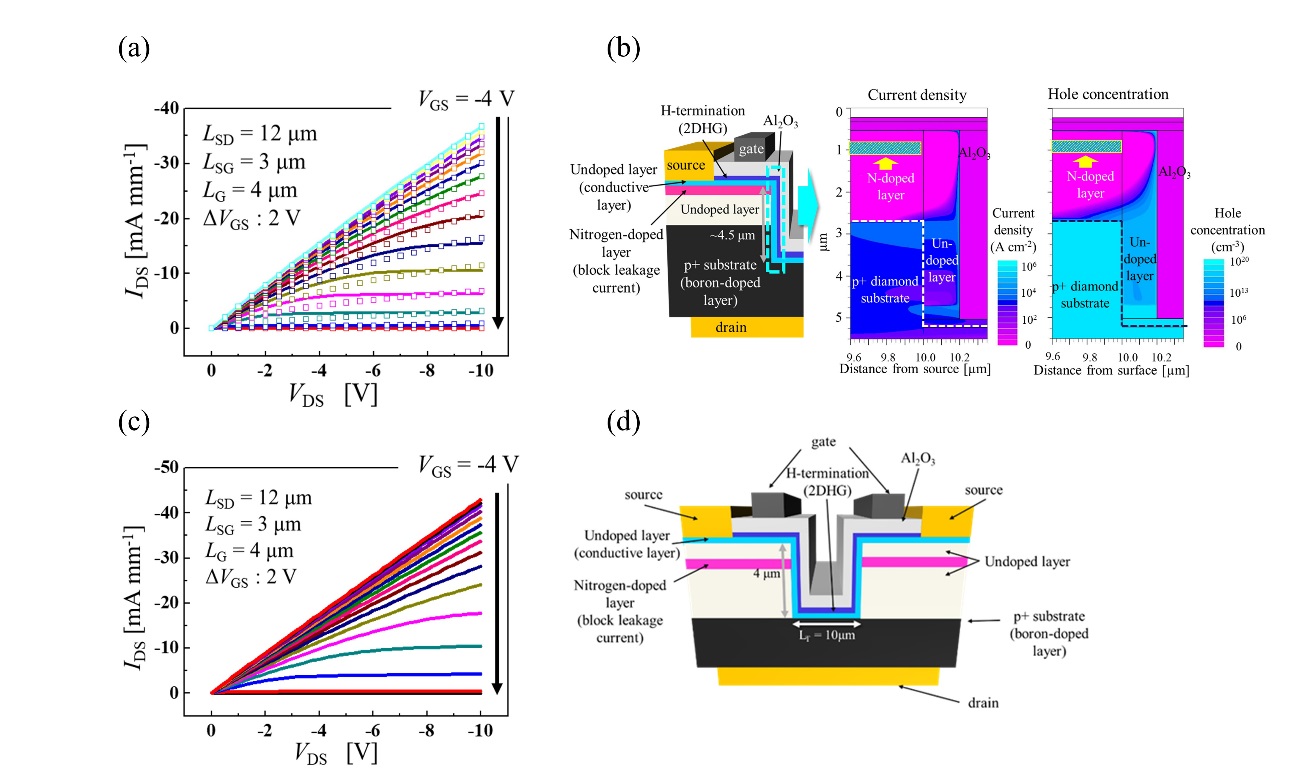


Figure. S4(a) *I*_DS_–*V*_DS_ characteristics of vertical-type device with N doped epitaxial layer of measured (plots) and simulated (solid line) results. (b) Total current density and hole concentration on the left side of trench. (c) *I*_DS_–*V*_DS_ characteristics of vertical-type device with trench that does not dig into the p+-type diamond substrate. (d) cross-section view of device use for (c)

Supplementary 5: Electric field simulation results (both kinds of devices).

Figure. S5(a) shows electric field simulation of vertical-type device with N implanted layer. From this figure, electric field concentrates near the trench sidewall rather than gate edge. This simulation results suggest that breakdown spot is not gate edge but trench sidewall (nitrogen-doped layer edge).

Figure. S5(b)(c) are enlarged view of electric field simulation of both kinds of vertical-type devices. Electric field concentrates at nitrogen-doped layer/undoped layer interface in both case, but electric field concentration is alleviated by increasing nitrogen-doped layer thickness.


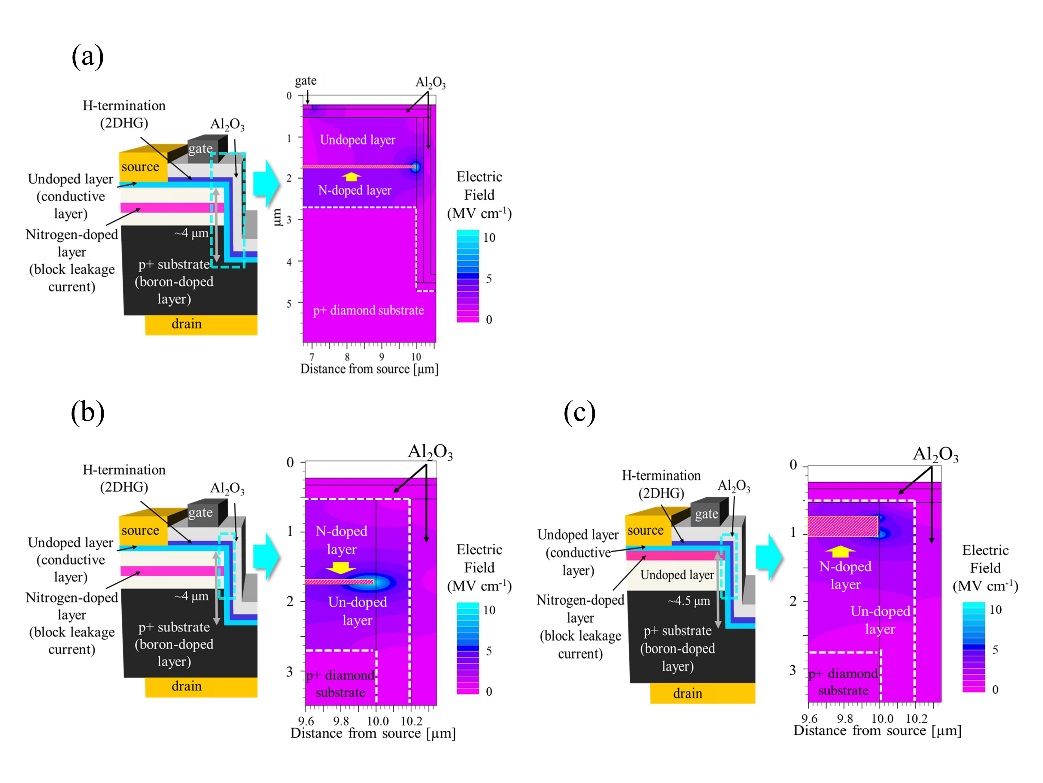


Figure. S5 Electric field simulation at trench structure.

(a) Electric field simulation of device with N implanted layer. (b) Enlarged electric field image with N implanted layer near trench structure. (c) Enlarged electric field image of device with N doped epitaxial layer near trench structure.

Supplementary 6: *I*_DS_-*V*_DS_ characteristics standardized by channel area.

Figure. S6 shows *I*_DS_-*V*_DS_ characteristics standardized by channel area (*W*_G_: 25 µm × *L*_SS_: 30 µm). The maximum current density is ~1480 A cm^-2^ and specific on-resistance is 31 mΩ cm^2^.


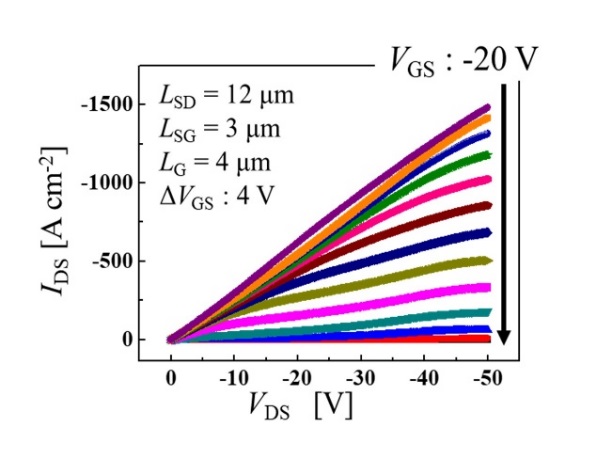


Figure. S6 *I*_DS_-*V*_DS_ characteristics of vertical-type device with N implanted layer standardized by channel area.

Supplementary 7: Reproduce of previous work by simulation.

In our previous work, vertical-type 2DHG diamond MOSFETs has large onset voltage (ref. 17). We confirm the cause of onset voltage by device simulation. We introduce thin nitrogen-doped layer at trench bottom because we consider that nitrogen-doped layer is not removed by etching process in previous work. Cross-sectional image and *I*_DS_-*V*_DS_ characteristics by simulation is shown in Figure. S7(a, b). Figure. S7(b) shows large onset voltage and this characteristic is similar to previous work (ref. 17), but current density is much higher. Carrier mobility and interface charge density in this simulation are the almost same as those of device with nitrogen-implantation layer.


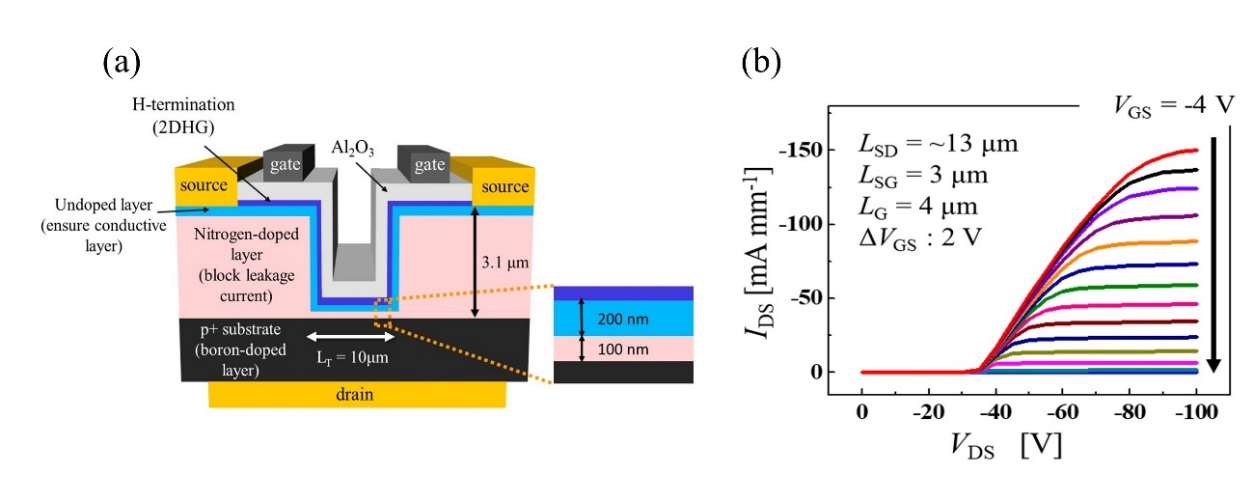


Figure. S7 Schematic diagram and *I*_DS_-*V*_DS_ characteristics by simulation.

(a) Cross-sectional image of vertical-type 2DHG diamond MOSFETs with thin nitrogen-doped layer at trench bottom. (b) *I*_DS_-*V*_DS_ characteristics of vertical-type device with nitrogen-doped layer at trench bottom.
